# “Mix of Mics”- Phenotypic and Biological Heterogeneity of “Multipotent” Muscle Interstitial Cells (MICs)

**Published:** 2012-10-17

**Authors:** Barbora Malecova, Pier Lorenzo Puri

**Affiliations:** 1 Sanford-Burnham Medical Research Institute, La Jolla, CA, USA; 2 Dulbecco Telethon Institute (DTI), IRCCS Fondazione Santa Lucia, Rome, Italy

**Keywords:** Stem cell, Multipotent, Skeletal muscle interstitium, Mesodermal lineage, Single cell analysis, Epigenetic

## Abstract

The capacity of adult skeletal muscle for regeneration appears to be limited, with progressive impairment in repair efficiency of injured muscles observed in chronic muscular disorders and during aging.

While satellite cells, the committed adult muscle stem cells, are the main direct cell source supporting the regenerative potential of adult skeletal muscles, the characterization of the cell types and signals that constitute the functional “niche” of satellite cells is currently the object of intense investigation. Recent studies have identified a functional relationship between satellite cells and various cell types located in key anatomical position, such as the interstitium of skeletal muscles. This heterogeneous population of muscle interstitial cells (MICs) appears to retain an intrinsic multipotency within the mesodermal lineage, and their direct or indirect contribution to myofiber turnover, repair and degeneration has been suggested by many studies that will be reviewed here. Given the existing gap of knowledge on lineage identity and functional properties of MICs, their detailed characterization at the single cell level holds the promise to provide key insight into the composition of this heterogeneous population and the dynamic transition through distinct sub-populations in healthy, diseased and aging muscles. This review provides an overview of the results of various studies describing the phenotype and the function of cells isolated from skeletal muscle interstitium, and discusses the importance of single cell transcription profiling in order to decipher the functional and phenotypical heterogeneity of muscle interstitial cells (MICs).

## Muscle Interstitial Cells (MICs): Functional niche for satellite cells and independent source of muscle progenitors?

Recent studies have revealed the existence of a population of cells in the interstitium of skeletal muscles that retain partial plasticity within mesoderm-derived lineages [1,2]. This anatomical property places them in the optimal position to sense mechanical or chemical perturbations of skeletal myofibers and transmit them to neighbor cells through diffusible signals. Likewise, the phenotypic plasticity of MICs appears to be dictated by environmental changes. As such, MICs can link and orchestrate the responses to physiological and pathological perturbations of muscle structure and function, including myotrauma, contraction, degeneration and metabolic changes. However, the nature of the functional interactions between these different cell types, and even their identity, are just beginning to be appreciated [3].

As most of MICs identified so far appear to be of mesodermal derivation and often originate from the vasculature of skeletal muscles, it is likely that MICs include heterogeneous populations of resident cell types and transiently amplifying cells derived from the vessels of injured or perturbed muscles. These cells establish functional interactions with satellite cells and other cell types (i.e. fibroblasts, immune infiltrate, myonuclei). Available studies have reported on distinct cell fates and functions adopted by MICs in different experimental conditions, ranging from fibro-adipogenic to myogenic lineages and activities [1,2,4,5]. Important issues that arose from these studies include the clarification of their physiological function during the transient activation upon acute injury versus their potential contribution to the pathogenesis of degenerative diseases caused by their chronic activation. Likewise, understanding the relationship between their activity and their cell fate - constitutive versus inducible – and the potential transition from one cell type to another during health, disease and aging, will pave the way for genetic and pharmacological manipulation of MICs for therapeutic purposes.

## MICs with Fibro-Adipogenic Potential

Two seminal discoveries from Rossi and Tsuchida labs have recently highlighted the potential contribution of a population of MICs endowed with a constitutive fibrotic and adipogenic fate to the regeneration or fibro-adipogenic degeneration of skeletal muscles [1,2]. Fibro-adipogenic progenitors (FAPs) were isolated by fluorescence-activated cell sorting (FACS) as CD45-/CD31- (lineage-negative lin-), stem cell antigen 1 (Sca1) and CD34 positive (lin-/Sca1+/CD34+), or lineage-negative, Sca1-positive and a7integrin (a7int)-negative (lin-/ Sca1+/a7int-) as by Rossi and colleagues [1]. Similarly, Uezumi et al. [2] isolated a functionally equivalent population of mesenchymal progenitor cells, as CD31–/CD45–/SM/C-2.6– cells that were positive for platelet-derived growth factor receptor alpha (PDGFRa) and beta (PDGFRb). A lineage overlap of these cells was indicated by the evidence that lin-/Sca1+/a7int- cells were positive for PDGFRa [1]. Thus, these are two seemingly equivalent cell populations that can collectively be indicated as FAPs.

FAPs reside in the interstitium of skeletal muscles and their differentiation potential and function seem to be dictated by the experimental conditions. When isolated from unperturbed muscles of wild type mice they differentiated *in vitro* with high efficiency into mature adipocytes, upon adipogenic culture conditions [1,2]; however, they can also generate osteoblasts upon bone morphogenetic protein 7 (BMP7) treatment, and smooth muscle-like cells when exposed to transforming growth factor beta (TGF-b) [2,5]. By contrast, they were unable to differentiate into skeletal muscle cells when cultured under myogenic differentiation conditions or transplanted into regenerating muscle [2]. Clonal analysis of these cells demonstrated that single mesenchymal progenitor cells can give rise to both adipocytes and collagen type-I-producing cells [5]. Similarly, Rossi and colleagues showed that FAPs display a bi-potency, consisting of an ability to differentiate into both perilipin-expressing adipocytes and alpha smooth muscle actin (a-SMA)-expressing fibroblast [1]. These lines of evidence reinforce the conclusion that FAPs [1] and mesenchymal progenitors [2] may be two very closely related multipotent cell populations with a strong fibro-adipogenic potential *in vitro* and partial plasticity within mesoderm-derived lineages.

What is the function of FAPs during muscle homeostasis and repair? Transplantation experiments clearly indicated that FAPs tend to adopt the adipogenic lineage *in vivo* under the effect of environmental cues. FAPs transplanted into healthy muscle do not support their engraftment neither their adipocytic differentiation *in vivo*, while FAPs support formation of ectopic fat when transplanted into degenerating muscle injected with the pro-adipogenic substance glycerol [1]. While no myogenic lineage was appreciated in FAPs, either cultured or transplanted, FAPs could support myogenesis indirectly – presumably via functional interactions with satellite cells. Indeed, FAPs enhanced endogenous muscle regeneration *in vivo*, and a promyogenic effect of FAPs on skeletal muscle differentiation was demonstrated also *in vitro* by co-cultures with primary myoblasts [1]. Diffusible factors are the candidate mediators of these functional interactions, although their identity has not been determined. Interestingly FAP proliferation is rapidly induced prior to satellite cells expansion, suggesting a role in the establishment of a pro-myogenic regenerative environment [1]. Similarly Uezumi et al. [2] observed that PDGFRa+ skeletal MICs significantly increased in number in cardiotoxin-induced regenerating muscle as well as in glycerol-injected degenerating muscle. Thus, it appears that transient amplification of FAPs supports early stages of muscle repair upon injury, with their ability to support muscle regeneration or ectopic fat formation being imparted by the environmental cues. At early stages of muscle regenereation, it is likely that FAPs promote satellite cell-mediated repair of injured muscles, presumably by providing the optimal environment for such process. Interestingly, direct contact of PDGFRa+ mesenchymal progenitors with myofibres inhibited adipogenesis of these cells [2], once again indicating that FAPs are regulated by the environment, and further emphasizing their functional plasticity in response to surrounding signals.

Further analysis of muscle-derived mesenchymal progenitors by Uezumi et al. [5] revealed that PDGFRa+ MICs treated with TGF-b isoforms show a dose-dependent induction of expression of the fibrosis-related molecules such as collagen type I and a-SMA. Fibrosis is characterized by excessive accumulation of extracellular matrix (ECM), with collagen type I being a main component of fibrotic ECM, and is the most deleterious outcome of many neuromuscular disorders, including Duchenne Muscular Dystrophy (DMD). Repeated cycles of muscle contraction-degeneration underlie DMD pathogenesis, leading to chronic activation of muscle regeneration and eventually resulting in the functional exhaustion of satellite cells and the ensuing fibro-adipogenic degeneration [6]. When regarded within the context of such chronic process, a persistent activation of MICs could impair their activity in support of muscle regeneration, and thereby bias their fate toward a constitutive fibro-adipogenic phenotype. While this is currently a speculation, it is interesting to note that TGF-b, which is highly expressed in DMD muscles [7] and significantly upregulated in fibrotic diaphragm muscle of *mdx* mice (the mouse model of DMD), promotes the expression of fibrosis marker in PDGFRa+ FAPs [5]. Taken together, these results suggest that FAPs might participate to the pathogenesis of DMD, as cellular targets and effectors of the cues skewing the destiny of diseased muscles toward fibro-adipogenic degeneration. Gene expression profiling identified number of genes differentially expressed in PDGFRa+ FAPs versus myogenic cells, after the TGF-b treatment [5]. This is an important step toward deciphering the gene networks operating in sub-populations of MICs implicated in fibro-adipogenic degeneration.

More recently, Dulauroy et al. [8] isolated and characterized a population of CD45-/CD31-/Gp38+ stromal cells that upon cardiotoxin-mediated muscle injury induced a disintegrin and metalloprotease ADAM12 expression. Interestingly, these cells also express PDGFRa and Sca1, and ADAM12+ cells give rise to a fibrogenic lineage *in vivo* and differentiate *in vitro* predominantly into a-SMA+ pro-inflammatory myofibroblasts when exposed to TGF-b-containing medium [8]. As such, they apparently share markers and biological features with FAPs. However, while ADAM12- population of lin-/ gp38+/PDGFRa+ cells show a strong adipogenic potential, ADAM12+ cells were very inefficient in differentiating into adipocytes, when placed in adipogenic medium. These distinctive features suggest that ADAM12+ cells indeed represent a small sub-population within the heterogeneous population of FAPs in skeletal muscle. *In vivo* during muscle injury, ADAM12+ cells expand in number and produce high amount of type I collagen. The pro-fibrotic fate of ADAM12+ cells is irreversible, and their presence in the tissue declines over the time, as the injured muscle heals, with ADAM12+ cell being actively eliminated from the healed tissue [8]. The functional overlap between lin-/gp38+/ PDGFRa+/ADAM12+ pro-fibrotic progenitors that are transiently amplified upon skeletal muscle injury and FAPs raises the question of whether they are transient fractions of an original population of MICs that are adopting a pro-fibrotic fate in response to TGF-b. Once again, detailed analysis of the transcriptome at the single cell level of MICs isolated in different laboratories will clarify biological and functional overlaps and differences, with the ultimate perspective of targeting them to control fibro-adipogenesis during muscle healing.

## MICs with Inducible Myogenic Potential

An increasing number of studies reporting on the myogenic potential of cells that originate from the vasculature of skeletal muscles and can adopt alternative mesoderm-derived cell fates suggests the possibility that these cells might actually represent sub-populations of MICs that retain an inducible myogenic potential. While these studies are summarized below, we anticipate that it is currently uncertain whether these cells are an independent source of muscle progenitors, if they transition into satellite cells or undergo unconventional myogenesis, and if they derive from a fraction of MICs in which the myogenic program is de-repressed under appropriate conditions – i.e. the regenerative environment.

Over a decade ago Gussoni et al. [9] reported on skeletal muscle-derived side population (SP) of cells isolated from wild type mice. SP cells were isolated based on their ability to efflux the fluorescent die Hoechst 33342, when compared to the main population (MP) of muscle-derived cells. They are typically positive for Sca1, either positive or negative for CD34, but negative for lineage markers and bone marrow markers (CD4, CD8, CD5, B220, Gr-1, CD11, c-Kit, CD45 and CD43). SP cells displayed a strong hematopoietic potential [9,10]. However, when transplanted intravenously into lethally irradiated *mdx* mice, SP cells could give rise to a dystrophin-positive donor cells in skeletal muscles [9]. An apparently similar population of muscle stem cells was later on described by Asakura et al. [11]. They isolated from skeletal muscles SP cells that showed distinctive features from satellite cells, including their localization outside the basal lamina of muscle fibers, with most of the cells being detected juxtaposed to blood vessels and platelet endothelial cell adhesion molecule (PECAM)- expressing endothelium. SP cells did not express the pro-myogenic markers paired box protein Pax7 and desmin, but expressed Sca1, and their myogenic potential was appreciated *in vitro* only by co-culture with myoblasts and *in vivo* upon intramuscular transplantation [11]. Furthermore, ectopic expression of MyoD could activate myogenesis in SP cells from Pax7 null mice [11], in which formation of satellite cells is typically deficient [11,12]. Collectively, these results indicated that SP cells are Sca1-expressing cells derived from the interstitium of skeletal muscle and are endowed with a constitutive hematopoietic potential and an ability to adopt the myogenic fate upon appropriate conditions (e.g. exposure to pro-myogenic signals). As such they could represent a fraction of MICs with an inducible myogenic potential. Tamaki et al. [13] also described CD34+ cells located in the interstitium of skeletal muscle that expressed the muscle regulatory basic helix-loop-helix (bHLH) factors MyoD and myogenin. These cells contributed to de novo formation of muscle fibers in skeletal muscles of postnatal rodents. As this population of skeletal muscle interstitial cells was positive for the expression of Sca1, but mostly negative for hematopoietic markers, and showed multilineage potential (i.e. myogenic, endothelial and adipogenic) by clonal analysis, it was proposed that these interstitial CD34+/Sca1+ cells could represent a subpopulation of the SP cells previously identified by Gussoni et al. [9], but endowed with more pronounced myogenic potential [9,14]. Zheng et al. [15] demonstrated the existence of another cell type, located in between human skeletal muscle fibers that co-expressed satellite cell and endothelial cell markers. These rare myo-endothelial cells account for about 1% of all Pax7+ satellite cells. The study reported that human muscle–derived endothelial cells (CD56–/CD34+/CD144+) and myoendothelial cells (CD56+/CD34+/CD144+) regenerate skeletal muscle upon intramuscular transplantation into immune-deficient mice injured by cardiotoxin more efficiently compared with myogenic cells (CD56+/CD34–/CD144–), and survive better under oxidative stress, as compared to myogenic CD56+ cells [15]. Transplanted myoendothelial cells were also located at the periphery of myofibers, suggesting that myoendothelial cells may replenish the satellite cell and/ or endothelial cell compartments [15]. Human myoendothelial cells also showed multipotency, as they could differentiate into osteogenic and chondrogenic lineage under specific culturing conditions [15]. The existence of multipotent Sca1+ muscle-derived stems cells with distinct properties than satellite cells, yet endowed with regenerative capacity, was also described by other groups [16,17]. However, these cells are located beneath the basal lamina of myofibers, and therefore are unlikely to represent a Sca1+ subpopulation of MICs.

Collectively, these works revealed the existence of distinct populations of cells derived from the interstitium of skeletal muscles that can adopt the myogenic lineage upon specific conditions – i.e. transplantation, co-culture with myoblasts or forced expression of MyoD. Their frequent endothelial derivation suggests that the intramuscular vasculature is a potential source of MICs that participate to muscle regeneration.

Indeed, Cossu’s lab isolated and characterized highly myogenic pericytes from human skeletal muscle biopsies [18]. In situ analysis showed that pericytes residing in skeletal muscle interstitium are associated with microvascular walls. Although pericyte-derived cells could differentiate into smooth muscle, osteoblasts and adipocytes under specific conditions, they also differentiated into skeletal myotubes with a very high efficiency when cultured in low serum-containing pro-myogenic medium, albeit with significantly different kinetics of myogenesis compared to satellite cells [18]. On the population level, pericyte-derived cells, unlike satellite cells, did not express Pax7, Myf5 and MyoD during proliferation, and up-regulated these pro-myogenic markers rapidly during differentiation, simultaneously with myogenin expression and shortly before appearance of MHC-positive myotubes [18]. Important feature of pericyte-derived cells isolated from skeletal muscle is their ability to repopulate muscles and contribute to *in vivo* myogenesis when transplanted systemically by intravenous injection into dystrophic immune-deficient mice [18]. This property makes pericyte-derived cells an ideal candidate for muscular dystrophy targeted therapeutic purpose. Of note, clinical trials based on transplantation of vessel-derived cell (mesoangioblasts) in DMD patents are currently under investigation [19,20]. Genetic labeling and *in vivo* tracing experiments in mice demonstrated that vessel-associated pericytes, but not endothelial cells, contribute *in vivo* to an early postnatal skeletal myogenesis and to the self-renewing Pax7+/Myf5- satellite cell pool [21]. Importantly, the regenerative muscle environment provided in dystrophic muscles or by cardiotoxin-mediated injury supports the contribution of endogenous pericyte-derived cells to postnatal skeletal myogenesis [21]. Consistently, a correlation between a significant increase in number of alkaline phosphatase (AP) positive pericytes in muscle biopsies from patients with muscular diseases, such DMD, and AP positive multinucleated muscle regenerating fibers [22] has been recently observed [23]. This study showed that AP expression by muscle fibers *in vivo* is exclusively dependent on fusion of pericytes [23], demonstrating that pericyte-derived cells are bona fide muscle progenitors distinct from satellite cells.

A population of human pericytes/perivascular cells that were similar to the pericytes described by Dellavalle et al. [18] was found associated with capillaries and arterioles in skeletal muscles as well as in number of different tissues by Peault and colleagues [24]. Human perivascular cells isolated from skeletal muscles and from non-muscle tissues, although not expressing myogenic markers myogenin, MyoD, Myf5, M-cadherin and Pax7 upon isolation, were highly myogenic *in vitro* when cultured in pro-myogenic medium as well as *in vivo* when intramuscularly transplanted into immune-deficient mice injured by cardiotoxin injection [24]. Phenotype of these cells closely resembled the phenotype of mesenchymal stem cells endowed with multilineage potential within mesodermal cell types when exposed to appropriate conditions [16,24].

Interestingly, in parallel with the identification of multipotent fibro-adipogenic progenitors FAPs [1] and mesenchymal progenitors [2] localized in muscle interstitium, Sassoon’s laboratory identified and characterized a population of muscle interstitial cells endowed with myogenic potential, but retaining the ability to differentiate into a-SMA-positive myofibroblasts [4]. These cells express the stress mediator PW1, but do not express the satellite cell marker Pax7 at the time of isolation. PW1+/Pax7- interstitial cells (PICs) were isolated based on FACS profile as lin-(CD45-/Ter119-) and CD34+/Sca1+. *In vivo*, intramuscularly injected PICs contributed very efficiently to skeletal muscle regeneration as well as to generating satellite cells and other PICs, suggesting an efficient self-renewal mechanism operating within this population [4]. Later work from the same group has revealed that PW1 expression can identify competent self-renewing stem cells in a wide array of adult tissues [25]. PICs isolated from muscles go through an intermediate state of co-expression of Pax7+/MyoD+ before forming skeletal muscle cells *in vitro,* and their myogenic potential is dependent on Pax7, while the smooth muscle fate was unaffected by Pax7 deletion [4]. Thus, unlike SP cells [11], the myogenic potential of PICs is Pax7-dependent [4]. Furthermore, failure of PICs to adopt a fibro-adipogenic phenotype and their ability to differentiate into skeletal muscle cells reveal striking differences between PICs and FAPs. However, it should be noted that PICs and FAPs share lineage markers (CD34 and Sca1) and the anatomical position (muscle interstitium), raising the possibility that PICs could represent a fraction of FAPs committed to alternative lineages.

## Lineage Switch in MICs and Potential Targets for Epigenetic Pharmacology

While the results reported in the above paragraphs clearly suggest the potential for lineage switch in MICs, with environmental conditions having a key role in dictating the specific lineage adopted, it is currently unclear the molecular mechanism that regulates such plasticity.

**Figure 1:**
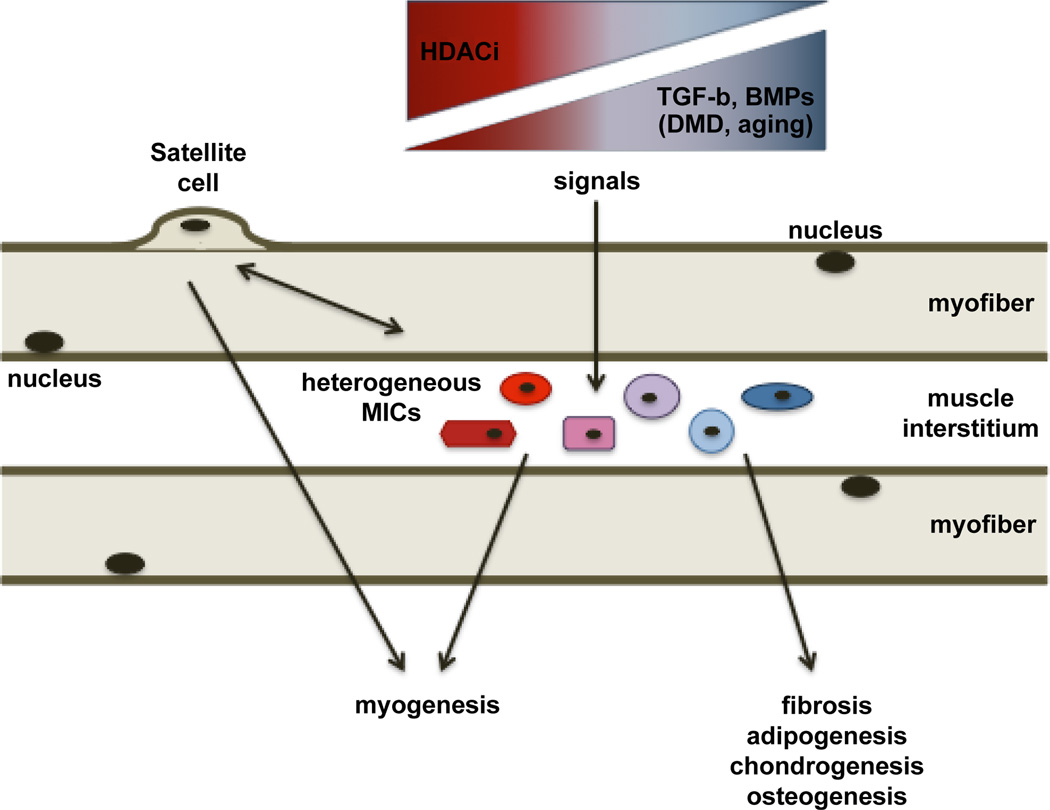
Different cell types located in the interstitium of skeletal muscles are depicted and indicated as muscle interstitial cells (MICs). These cell types share a partial pluripotency within the mesoderm-derived lineages and can contribute to the repair of skeletal muscles directly or indirectly. Indirect interactions with other cell types, and in particular with the main cellular effectors of muscle regeneration (the satellite cells), has been shown to promote early regeneration events in injured muscles, possibly via paracrine signals. Given their potential to adopt a fibro-adipogenic or myogenic phenotype in response to environmental cues, MICs can be a pivotal cellular component that determines whether muscle healing occurs by regeneration or fibroadipogenic degeneration. As such, MICs could contribute to the pathogenesis of chronic degenerative muscular diseases, as well as to age-dependent decline of muscle mass and activity (sarcopenia). Signaling pathways, such as transforming growth factor beta (TGF-b) and bone morphogenetic protein (BMP) signallings, that promote degenerative outcome of failed skeletal muscle regeneration during aging and Duchenne Muscular Dystrophy, are depicted. Given the phenotypic and functional heterogeneity of MICs, the identification of sub-populations and their dynamic transitions in response to developmental, physiological and pathological signals, based on transcriptome analysis at the single cell level, will clarify their function and identify potential target for their pharmacological manipulation, for example by HDACi treatment that can reverse skeletal muscle degeneration [33].

The transition from one lineage to another is typically regulated at the gene expression level, with the simultaneous activation and repression of transcription of discrete subset of genes guiding the selection of a specific lineage. Epigenetic control of gene expression has recently emerged as the fundamental mechanism of regulation of cell fate in embryonic and adult stem cells [26–8]. Epigenetic silencing of alternative cell fates can be permanent or reversible. Therefore, epigenetic drugs are interesting candidates to pharmacologically manipulate the lineage of multipotent mesoderm-derived cells for therapeutic purposes. For instance, histone deacetylase (HDAC)-mediated repression of the myogenic program in undifferentiated myoblasts has been extensively reported [6,29,30], and the ability of epigenetic drugs, such as HDAC inhibitors (HDACi), to promote skeletal myogenesis has been shown *in vitro* and *in vivo* [31,32]. These studies demonstrated that changes in muscle environment influence the ability of HDACi to promote regeneration of skeletal muscles in a stage-dependent manner [31,32]. Importantly, in the context of dystrophic muscles in their regenerative stage (young *mdx* mice) HDACi promote endogenous regeneration and repress fibrosis [33] (Figure 1). While, it is currently unknown how HDACi can simultaneously achieve such an effect, it is possible that these drugs can target muscle resident cells endowed with partial pluripotency (e.g. some of the MICs reported above) in which lineage commitment is reversibly repressed by HDACs. Thus, the combination of environmental cues and pharmacological manipulation of the epigenetic control of gene expression are exploitable conditions for future applications of epigenetic pharmacology in regenerative medicine.

## Single Cell Profiling as a Tool to Tackle Heterogeneity of Multipotent Progenitors

The evidence available from the studies reviewed above reveals certain common as well as distinct characteristics of MICs, and it is likely that sub-populations of muscle interstitial cells isolated and characterized in different laboratories may be in some instances largely overlapping.

An intriguing feature of majority of mononucleated cells residing in skeletal muscles is their multipotency and their ability to readily differentiate into chondrocytic, fibrotic, adipocytic, osteogenic, smooth muscle-like and myogenic lineage under specific conditions generated by the environment.

Molecular characterization of alternative populations of MICs, which are highly responsive to external cues, will be essential to answer fundamental questions on skeletal muscle regeneration. MICs are a heterogeneous population, whereby each cell type is regulated by multiple signaling pathways and expresses specific subsets of genes that serve as a “blueprint of identity”. Sub-populations of MICs with specific characteristics receive and interpret the cues from the regenerating environment to adopt specific cell fates within mesodermal lineage. An important question to address in the future regards the elucidation of the components of the environment that modulates the fate of MICs. However, the natural heterogeneity of MICs compromises efforts aimed at understanding their physiology and function. In molecular biology, the gene expression levels are by default measured on large pools of cells. Cell population measurements will not reveal how a particular gene transcript is distributed among the individual cells, as bulk measurements easily underestimate potentially important gene correlations. Instead, single cell analysis of gene expression profiling is a more effective strategy to couple transcriptional regulation and signal networks in dynamic cell sub-populations [34–6] and has been successfully applied in number of studies that characterized specific functional fractions of heterogeneous cell populations [35,37–44], reviewed in [45].

One outstanding example of how to use single cell analysis was offered by recent studies on the characterization of cell types that contribute to mammalian brain development. Neural progenitor cells are classically categorized into two distinct cell types, the apical progenitors, which can function as a stem-like undifferentiated progenitor cell, and the basal committed progenitor cell. The heterogeneity of neural progenitors, the mechanism by which apical progenitors differentiate into basal progenitors, and the way these neural progenitors are controlled during cortical development, are unclear. Single cell genome-wide gene expression by microarray-based profiling of single neural progenitors in mid-embryonic stage mouse dorsal forebrain identified four sub-classes of cells, and revealed two distinct classes of neural progenitors in mouse embryonic cerebellum, corresponding to apical and basal neural progenitors [39]. Importantly, this study also identified that Notch signaling is an important player in the heterogeneous division patterns of these progenitor cells and in self-renewal of apical progenitors into nascent basal progenitors [39]. This is an example of the power of genome-wide single cell gene expression profiling of heterogeneous cellular populations and how this technology can lead to the identification of signaling pathways influencing the fate of distinct cellular sub-populations.

Another elegant example of using the single cell transcriptome analysis with RNA-seq technology is offered by recent work from Surani’s lab. To gain insight into the precise changes accompanying the process of conversion of inner cell mass (ICM) to embryonic stem cells (ESCs), the authors used blastocysts from Oct4-DPE-GFP transgenic mice where Oct4-DPE-GFP reporter is under the control of the distal Oct4 enhancer. The study provides insight into the dynamic molecular changes that accompany cell-fate changes. During the conversion of ICM cells to ESCs, there is an arrest of the developmental program, which is subverted *in vitro* to favor unrestricted self-renewal, while retaining the ability to undergo differentiation into various cell types [38]. Such study paves the path for studying the regulation and differentiation of adult multipotent progenitors, and demonstrates that, when combined with lineage tracing technology, wide-genome analysis of the transcriptome at the single cell level has the potential to dissect the fate of single progenitor cells throughout the development and identify the regulatory events underlying this process.

A comprehensive picture of the network of signaling pathways and gene interactions involved in regulation of physiology of adult muscle progenitors at the level of the single-cell gene expression profiling of muscle interstitial cells MICs will allow us to decipher the molecular networks underlying regulation of lineage commitment of MICs in regenerating and degenerating skeletal muscle tissue. Understanding the biology of the cells responsive to the environmental cues that affect muscle regeneration provides the ground for therapeutic interventions that would shift a balance between muscle regeneration and fibroadipogenic degeneration in muscular dystrophy.

## Abbreviations

ADAM12: A Disintegrin and Metalloprotease 12
AP: Alkaline Phosphatase
a-SMA: alpha Smooth Muscle Actin
BMP7: Bone Morphogenetic Protein 7
DMD: Duchenne Muscular Dystrophy
FACS: Fluorescence-activated Cell Sorting
FAPs: Fibro-adipogenic Progenitors
HDAC: Histone Deacetylase
HDACi: HDAC inhibitor
ICM: Inner Cell Mass
MICs: Muscle Interstitial Cells
PDGFR: Platelet-derived Growth Factor Receptor
PICs: PW1-positive Interstitial Cells
PECAM: Platelet Endothelial Cell Adhesion Molecule
SCA1: Stem Cell Antigen 1
TGF-b: Transforming Growth Factor Beta
